# Supplementary material for: Neoadjuvant antiangiogenic therapy reveals contrasts in primary and metastatic tumor efficacy
Source: EMBO Mol Med. 2014 Oct 31;6(12):1561–76. doi: 10.15252/emmm.201403989 (PMC4287975; doi:10.15252/emmm.201403989)
Supplement: Supplementary file 1 — Supplementary Information [file emmm0006-1561-sd1.pdf]

Supplemental Information:

Table of Contents

Supplemental Materials and Methods: 3
Cell lines: 3
Drugs and Doses Used: 3
Tissue Processing and Immunostaining 3
Tumor Tissue Microarray (TMA) 4
Tissue Image Acquisition and Quantification 4
Evaluation of end-stage metastatic disease distribution 4
Bioluminescent Imaging 5
Statistical Analysis 5
Supplemental Results: 6
Defining an optimal window for neoadjuvant therapy depends on surgical timing and tumor cell-specific metastatic potential 6
Distribution of metastasis varies depending on neoadjuvant therapy 7
Molecular marker changes in primary tumor following neoadjuvant therapy may predict for post-surgical benefit 7
Supplemental Figure Legends 8
Figure S1: Neoadjuvant therapy window determined by evaluating metastatic potential and optimal surgical time 8
Figure S2: Primary tumor response to neoadjuvant sunitinib treatment is not predictive of post-surgical survival in a model of melanoma metastasis. A 8
Figure S3: Modulating neoadjuvant Oxi4503 dose and surgical timing can improve post-surgical survival. 8
Figure S4: Survival curves for Figure 4A. 9
Figure S5: Survival curves for Figure 4B 9
Figure S6: Metastatic disease distribution following neoadjuvant therapy depends on model and mode of VEGF pathway inhibition. 9
Figure S7: Elevated primary tumor Ki67 expression following neoadjuvant therapy may predict post-surgical survival benefit depending on treatment and metastasis model. 9
Figure S8: Randomization and equal sorting of mice prior to neoadjuvant treatment. 9
Supplementary Tables and Figures 11
Table S1 11
Figure S1 12

Figure S2 13
Figure S3 14
Figure S4 15
Figure S5 16
Figure S6 17
Figure S7 18
Figure S8 19
Reference list 20

Supplemental Materials and Methods:

Cell lines:

The human LM2-4 cells are a metastatic variant of the MDA-MB-231 breast cancer-cell line derived after two rounds of *in vivo* lung metastasis selection in mice (Munoz et al, 2006). SN12-PM6 are a metastatic variant of the SN12C human kidney cancer cell line previously selected after multiple rounds of in vivo selection (a kind gift from I.J. Fidler ,MD Anderson) (Fidler et al, 1990). LM2-4^{LUC+} and SN12-PM6^{LUC+} are variants, the former co-transfected with plasmids expressing the firefly luciferase gene (pGL3-control, Promega Corporation, Madison, WI, USA) and neomycin resistance gene (C. Jedeszko, unpublished data), the latter with a single plasmid bearing both genes as previously described (Ebos et al, 2008). WM113/6-4L is a variant of the human melanoma WM113 line isolated *in vivo* for its ability to metastasize to the lung (Cruz-Munoz et al, 2008). Mouse kidney RENCA^{LUC+} expressing luciferase have been described previously (a kind gift from R.Pili, Roswell Park Cancer Institute) (Kato et al, 2007). All human cells, including the human melanoma MeWo cells (obtained from the American Type Culture Collection, Manassas, VA), were maintained in Dulbecco's modified Eagle's medium (HyClone, Cat. #SH30022.01) with 5% heat-inactivated fetal bovine serum (HyClone, Cat. #SH30396.03). Mouse RENCA^{LUC+} cells were maintained in Roswell Park Memorial Institute (RPMI) medium (Corning cellgro, Cat. #10-040-CV) with 10% heat-inactivated fetal bovine serum. All cells were incubated at 37°C and 5% CO_2 in a humidified incubator.

Drugs and Doses Used:

SU11248/sunitinib malate (Sutent®©, Pfizer) and AG013736/Axitinib (Inlyta®©, Pfizer) were suspended in Vehicle formulation containing carboxymethylcellulose sodium (USP, 0.5% w/v), NaCl (USP, 1.8% w/v), Tween-80 (NF, 0.4% w/v), benzyl alcohol (NF, 0.9% w/v), and reverse osmosis deionized water (added to final volume) and adjusted to pH 6 - as described in (Ebos et al, 2007). Drug aliquots were prepared once weekly and kept in the dark at 4°C. UFT, a 5-fluorouracil pro-drug (Taiho, Japan) was diluted in 0.1% hydroxypropylmethylcellulose vehicle (Taiho, Japan) and gavaged daily. Anti-VEGF antibodies G6.31 and B20 (Genentech, Roche), and anti-VEGFR-2 CT322 (Adnexus, Waltham, MA) were diluted in sterile PBS before intraperitoneal (i.p). injection. Cyclophosphamide (CTX) (Baxter Oncology GmbH, Mississauga, Ontario, Canada) was diluted with sterile normal saline, and administered i.p. at the maximum tolerated dose (MTD) or as a low dose metronomic (LDM) regimen through the drinking water, as previously published (Man et al, 2002). OXI4503, a vascular disrupting agent obtained from Oxigene (San Francisco, CA), was prepared in sterile normal saline and delivered i.p. injectable vinblastine sulphate (VBL) was diluted in normal saline and administered i.p. CTX and VBL were purchased from the institutional pharmacy (Sunnybrook Research Institute). All doses and treatment durations are summarized in Supplementary Table 1.

Tissue Processing and Immunostaining

Excised kidneys were immediately placed in Tissue-Tek optimum cutting temperature compound (Miles Inc., Elkhart, IN), frozen in dry ice, and then kept at -70°C. Simultaneously, separate tissues were fixed in 10% zinc-buffered formalin (Z-FIX, Anatech Ltd., Battle Creek, MI, USA) for 24 hours, then stored in 70% ethanol. Five micron-thick frozen sections were fixed in 4%

paraformaldehyde, blocked with protein block serum-free solution (Dako; x0909), and stained with rabbit monoclonal anti-human Vimentin antibody (1:70 dilution; ab16700; Abcam, Cambridge, MA, USA) to distinguish between mouse and human tumor tissue, and rat anti-mouse CD31 antibody (1:25 dilution; DIA-310; Dianova) to visualize endothelial cells, followed by a FITC-conjugated anti-rabbit antibody (1:200 dilution; 554020; BD Pharmingen) and a Cy3-conjugated anti-rat antibody (1:200 dilution; A10522; Invitrogen). All antibodies were diluted in background-reducing components solution (S3022; Dako). Sections were counterstained with DAPI (1 μg/ml dilution, Molecular Probes) and mounted with Vectashield hard set mounting medium (H-1400; Vector). Staining with mouse IgG2a (DakoCytomation, Carpinteria, CA) was used as a negative control. Five micron paraffin-embedded tissues were deparaffinized and rehydrated using a xylene-ethanol gradient, and subjected to detection and counterstaining with haematoxylin and eosin (H&E) by the Mouse Tumor Model Resources core at Roswell Park Cancer Institute.

Tumor Tissue Microarray (TMA)

Resected melanoma tumors were cut such that the outer, viable portion of the tumor, including a section of skin, was taken and mounted in paraffin. Three, 0.6 mm-wide cylindrical biopsies were bored from each tumor portion using a Manual Tissue Microarrayer, Model MTA-I (Beecher Instruments, Inc., Sun Prairie, WI), and mounted in another pre-bored paraffin block to create a tumor tissue microarray slide. Cut sections were deparaffinized and rehydrated using a xylene-ethanol gradient. Target antigen retrieval was performed by boiling in sodium citrate buffer, pH 6.0. Slides were stained with rabbit monoclonal anti-human Vimentin antibody (1:70 dilution; ab16700; Abcam, Cambridge, MA, USA), rat anti-mouse CD31 antibody (1:25 dilution; DIA-310; Dianova), or rabbit anti-human ki67 antibody (1:500 dilution; RM-9106-S0; Thermo Scientific) using the EnVision G|2 Doublestain system (K5361; Dako) for Vimentin and CD31 double staining, or the Histostain® SP Broad Spectrum kit (959643; Invitrogen) for Ki67 staining.

Tissue Image Acquisition and Quantification

Frozen sections were visualized under a Carl Zeiss Axio Imager A2 fluorescence microscope, using FITC (470 nm), Cy3 (550nm) and DAPI (350 nm) fluorescence filters. Images were captured with a Zeiss Axiocam camera connected to the microscope using AxioVision 2.8 software. Tissue sections were imaged at 25, 100 or 400X (2.5, 10 or 40X objective/10X eyepiece). Paraffin and TMA sections were scanned with ScanScope XT System (Aperio Technologies) using ImageScope software (Aperio Technologies). Quantification of intensity was performed with imageJ. For the TMA sections, images were analyzed all at once using macro functions. Each stain was separated with the colour deconvolution plugin and relative intensity was measured for each dye (DAB, Permanent Red, and Haematoxylin). For frozen sections, vessel number ($CD31^+$ cells) slides were randomized and quantified visually.

Evaluation of end-stage metastatic disease distribution

Animals were sacrificed according to institutional guidelines, with endpoints including signs of distress, labored breathing, weight loss, etc., and post-surgical evaluation of spontaneous metastatic disease was conducted by necropsy. Metastatic disease distribution was scored

visually as present (+) or absent (-) in organs and lymph nodes following a predetermined guideline (Tracz et al, 2014). End-stage metastatic disease distribution scoring and summary scoring (See Figure 1I and Figure S7) is independent of overall efficacy. Animals not necropsied were included in the overall survival analysis but excluded from analysis of disease distribution.

Bioluminescent Imaging

Bioluminescent imaging was performed with a highly sensitive, cooled CCD camera mounted in a light-tight specimen box (IVIS™; Xenogen, Alemeda, CA) as previously described (Ebos et al, 2009). Briefly, mice were injected intraperitoneally with substrate D-luciferin at 150 mg/kg in Dulbecco's Phosphate Buffered Saline (Invitrogen, Carlsbad, CA, USA) and anesthetized (4% isoflurane in oxygen for induction, 2% for maintenance) after a 10 minute interval. One to five mice were then placed onto the warmed stage inside the light-tight camera box with continuous exposure to 1–2% isoflurane and imaged for 1 minute. Light emitted from the bioluminescent cells was detected by the IVIS™ camera system with images quantified for tumor burden using a log-scale color range set at $5x10^4$ to $1x10^7$ for the xenograph models and $5x10^5$ to $1x10^7$ for the syngeneic model and measurement of total photon counts per second (photons/sec) using Living Image® software (Xenogen).

Statistical Analysis

In addition to the statistical analysis described in the Methods and Materials section, Kaplan-Meier analysis was used for comparing high levels of protein expression to low levels to survival outcomes (Supplemental Figure S7). Unpaired two-tailed Student t-tests were used to compare protein expression levels.

Supplemental Results:

Defining an optimal window for neoadjuvant therapy depends on surgical timing and tumor cell-specific metastatic potential

To evaluate preclinical neoadjuvant therapy in mice, we first undertook experiments to identify an optimal therapeutic window to compare drug effects on pre-surgical ‘primary’ tumor growth to post-surgical ‘secondary’ spontaneous metastatic spread and survival. We evaluated three parameters critical to a neoadjuvant therapy tumor model system. First, we compared the ‘metastatic potential’ (MP) of three different human tumor cell lines, each of different origin (melanoma, breast, and kidney), which allowed identification of the optimal tumor size prior to surgery necessary to ensure sufficient metastatic disease. Second, we established an optimal surgical time (OST), which was used to define a tumor growth period needed to maximize spontaneous metastasis for neoadjuvant therapy study. This is critical to consider because surgery too early would minimize (or eliminate) MP and surgery too late would lead to localized invasion and confound spontaneous (distant) metastatic growth. Third, we defined a preclinical measure of residual cancer burden (RCB) at time of resection, i.e., the amount of detectable disease remaining at time of surgery. This allowed for potential comparisons with clinical parameters of pCR and monitoring of surgical variability (see methods for details). MP and OST were assessed using variables of tumor burden (e.g., volume and weight) and time of tumor growth (e.g., number of days from implantation to surgery). Four tumor models of spontaneous metastasis were used, including human breast (LM2-4^{LUC+}) (Ebos et al, 2008), human melanoma (WM113/6-4L) (Cruz-Munoz et al, 2008), human kidney (SN12-PM6^{LUC+}), and mouse kidney (RENCA^{LUC+})(Tracz et al, 2014) cells. Resection of orthotopic breast and melanoma tumors, as well as RENCA^{LUC+}-bearing kidneys at varying timepoints showed a negative correlation between pre-surgical primary tumor burden and post-surgical survival. For LM2-4^{LUC+} and WM113/6-4L resected tumor size was measured (Figure S1A - R^2=0.6106 and P=0.0001; Figure S1B - R^2=0.019 and P=0.6098), respectively. For RENCA^{LUC+}, SN12-PM6^{LUC+}, gross kidney weight and bioluminescence (BL) were used with significance observed for both in BL (Figure S1C - R^2=0.1479 and P=0.0491; Figure S1D - R^2=0.4748 and P=0.0092, respectively). A minimum OST threshold (i.e., the tumor weight or kidney weight where some mice were cured by surgery and thus not useful in neoadjuvant treatment testing) was established as 0.57-0.45g for LM2-4^{LUC+}; 0.328-0.073g for WM113/6-4L, and <0.1g for RENCA^{LUC+} (Figure S1A, S1B, and S1C - grey bars). In the SN12-PM6^{LUC+} kidney tumor model, a minimum OST was not established in the predetermined timeframe necessary to conduct a neoadjuvant treatment comparison (approx.7-14 days). OST for the LM2-4^{LUC+},, WM113/6-4L , and RENCA^{LUC+} models were between 18-30 days (Figure S1E), 34-38 days (Figure S1F), and 20-26 days (Figure S1G), respectively. Ranges of acceptable RCB - including localized disease (shown only by bioluminescence) - are shown in Figure S1F (LM2-4^{LUC+}and SN12-PM6^{LUC+}) (see methods for details). Taken together, our results show that MP was influenced by tumor size and growth rate, and varied between tumor models. Similarly, parameters of OST and RCB are critical to define for clinically relevant evaluation of neoadjuvant therapy.

Distribution of metastasis varies depending on neoadjuvant therapy

We have previously determined that short-term sunitinib preconditioning in tumor-free mice prior to tumor cell inoculation in an experimental metastasis model lead to an increase in metastasis, but also that pre-treatment did not alter the metastatic distribution patterns compared to control (Ebos et al, 2009). Here we sought to assess metastatic disease distribution after multiple neoadjuvant therapies and regimens by evaluating where metastatic disease could be visualized at individual mouse endpoint. This measurement is independent of treatment efficacy. For the neoadjuvant studies performed with LM2-4^{LUC+} breast cells in Figure 6B, we visually scored metastasis presence or absence at necropsy and compared heatmap data (values compared to control group) to evaluate treatment influence (Figure S6). No consistent trends were observed that suggest neoadjuvant treatment influenced a preferred location of eventual metastasis compared to vehicle treated controls.

Molecular marker changes in primary tumor following neoadjuvant therapy may predict for post-surgical benefit

We used histopathological analysis of primary tumors following neoadjuvant therapy to examine whether potential correlations could be observed with overall (postsurgical) survival. Following neoadjuvant studies described in Figures 3C and 5C, core samples were taken from primary human WM113/6-4L melanoma tumors to generate tumor microarrays (TMAs). These TMAs were stained for expression of CD31, human vimentin, and Ki67 – a marker of proliferation (Figure S7A). Kaplan-Meier survival analysis for all treatment groups showed high expression levels of ki67 (>2.21 scores), CD31 (>2.84 scores), and Vimentin (>1.40 scores) did not correlate with increased survival (Data not shown). However, analysis of individual treatments showed that elevated Ki67 levels (>2.21) following B20 and CT322 treatment predict for decreased survival (Figures S7B and S7C), whereas the opposite was observed following sunitinib treatment as elevated ki67 levels predicted for prolonged survival. Interestingly, similar correlations with CD31 were not observed following in SN12-PM6^{LUC+} kidney models that were non-responsive to neoadjuvant sunitinib treatment described in Figure 1A-D (see Figure S7D, S7E, S7F). Taken together, these results suggest that intratumoral Ki67 levels at time of surgery following neoadjuvant antiangiogenic therapy may have the potential as a biomarker for predicting overall survival however this may be dependent on the type of VEGF pathway inhibitor used, with extracellular and intracellular inhibitors potentially yielding opposite results.

Supplemental Figure Legends

Figure S1: Neoadjuvant therapy window determined by evaluating metastatic potential and optimal surgical time._A) Following orthotopic (intra-mammary fat pad) implantation of $2x10^6$ LM2-4^{LUC+} human metastatic breast carcinoma cells, tumors were surgically excised after 16, 17,18,30, 34, 36 and 37 days (4 separate experiments, N=33 mice total) and weights were compared to overall survival. **B)** $1x10^6$ WM113/6-4L human metastatic melanoma cells implanted orthotopically (intra-dermally), resulting tumors were surgically excised at days 22, 27, 34, 41, and 44 (2 separate experiments, N=16 mice total) and weights were compared with overall survival. **C)** $4x10^4$ $Renca^{luc+}$ mouse kidney carcinoma cells implanted orthotopically (sub-capsular space), resulting encapsulated tumor bearing kidneys were surgically removed 10, 18, 21, 26, 30 days after implantation (N=26) and resected tumor weights or BLI were compared with overall survival. **D)** $2x10^6$ SN12-$PM6^{LUC+}$ human metastatic kidney carcinoma cells implanted orthotopically (sub-capsular space), resulting encapsulated tumor-bearing kidneys were surgically removed on day 27 (N=13 mice total) and excised kidney weight or BLI were compared with overall survival. **E-G)** Determination of OST for neoadjuvant therapy by comparing early 'curative' tumor removal timepoints (i.e., no postsurgical metastasis) to late 'non-neoadjuvant' tumor removal timepoints (i.e., local invasion and obvious metastasis). **E)** LM2-4^{LUC+} OST was between 18-30 days (mean = day 24). **F)** WM113/6-4 OST was after 34 days (macroscopically local invasion not observed in studies conducted). **G)** $Renca^{LUC+}$ OST was between 18-26 days, with preference for earlier time-points to reduce probability of invasive disease. **H)** Representative examples of an acceptable range of RCB, including distant and local post-surgical disease measured by BL (breast model-left panel; kidney model-right panel). Grey bars in panels A-C, indicates primary tumor weights from animals that did not develop metastasis and therefore represents a minimum threshold of tumor growth necessary to evaluate neoadjuvant therapy; OST, Optical Surgical Time; RCB, Residual Cancer Burden; BLI, bioluminescence imaging.

Figure S2: Primary tumor response to neoadjuvant sunitinib treatment is not predictive of post-surgical survival in a model of melanoma metastasis. A) Comparison of tumor volumes in SCID mice bearing orthotopic human WM113/6-4L melanoma tumors receiving neoadjuvant sunitinib for 14 days. **B**) Corresponding quantification of resected WM113/6-4L tumor weights (**left panel**) following neoadjuvant sunitinib treatment cessation with representative images shown (**right panel**) **C)** Post-surgical survival (WM113/6-4L model). *Symbols and bars for box and whiskers plot:* median (line), upper/lower quartile (box), min/max (error bars). *Survival analysis:* Hazard ratio (HR), confidence interval (CI), overall survival (OS) based on Kaplan-Meier or Cox regression analysis. N=8-12 mice per group. Neoadj. Tx, Neoadjuvant treatment.

Figure S3: Modulating neoadjuvant Oxi4503 dose and surgical timing can improve post-surgical survival. A) SCID mice implanted with LM2-4^{LUC+} human breast cancer cells in the mammary fat pad and treated with vehicle or OXI4503 (10 mg/kg/day for 14 days). Comparison of tumor volume by caliper measurement shown. **B**) From study in (A), comparison of tumor weight following surgery (36 days post-implantation), **C)** From study in (A), post-surgical survival following neoadjuvant OXI4503 treatment. **D)** SCID mice implanted with LM2-4^{LUC+} human breast cancer cells in the mammary fat pad and treated with vehicle or OXI4503 (50 mg/kg/day) for 7 days . Comparison of tumor volume by caliper measurement shown. **E**) From study in (D), comparison of tumor weight following surgery (30 days post-implantation). **F)**

From study in (D), post-surgical survival following short-term (high-dose) OXI4503 treatment compared to control. **G)** Comparison of post-surgical survival following short-term (high-dose) OXI4503 treatment compared to short-term (low-dose) OXI4503 treatment. *Symbols and bars for box and whiskers plot*, median (line), upper/lower quartile (box), min/max (error bars). *Survival analysis:* Hazard ratio (HR), confidence interval (CI), overall survival (OS) based on Kaplan-Meier or Cox regression analysis. N=8-9 mice per group. ** p<0.01 | *** p<0.001 compared to control.

Figure S4: Survival curves for Figure 4A. Treated groups were compared to vehicle. Treatments include: **A)** sunitinib (120 mg/kg), **B)** DC101, **C)** G6.31, **D)** B.20, **E)** CT322, **F)** MTD CTX, **G)** OXi4503, **H)** XRT, **I)** LDM CTX, **J)** LDM UFT, **K)** LDM CTX/UFT. Hazard ratio (HR) and confidence interval (CI) based on Kaplan-Meier or Cox regression analysis. * p<0.05 | *** p<0.001 compared to control.

Figure S5: Survival curves for Figure 4B. Treated groups were compared to vehicle. Treatments include: **A)** sunitinib (120 mg/kg), **B)** LDM CTX/UFT, **C)** PF10966, **D)** OXi4503, **E)** DC101, **F)** CT322, **G)** G6.31, **H)** B.20. Hazard ratio (HR) and confidence interval (CI) based on Kaplan-Meier or Cox regression analysis. * p<0.05 | *** p<0.001 compared to control.

Figure S6: Metastatic disease distribution following neoadjuvant therapy depends on model and mode of VEGF pathway inhibition. A) For neoadjuvant studies described in Figure 6 for the breast (LM2-4^{LUC+}) model described in Figure 6B, metastatic disease presence or absence was scored at endpoint (necropsy) and used to generate a heatmap to show differential disease distribution patterns. Scoring indicates fold-change compared to control, with increases (red) and decreases (blue) shown.

Figure S7: Elevated primary tumor Ki67 expression following neoadjuvant therapy may predict post-surgical survival benefit depending on treatment and metastasis model. A-C) Experiments performed using tissue taken from primary WM113/6-4L melanoma tumors excised after neoadjuvant therapy from studies described in Figure 3C and Figure 5C. **A)** Representative Tissue MicroArray (TMA) examples of neoadjuvant treated tumors stained with Ki67 or co-stained with CD31 and vimentin. **B)** Animals in each treatment group where separated based on the expression levels of ki67, CD31, or vimentin (above and below) to compare primary tumor molecular changes as potential predictive markers for post-surgical survival. **C)** Log-rank p-values of Kaplan-Meier survival analysis for each treatment group compared to expression levels for CD31, Vimentin, and ki67. Significant values are shown in bold red color. **D-F)** Experiments performed using kidneys bearing SN12-PM6^{LUC+} tumor resected following neoadjuvant sunitinib therapy from Figure 1A-D. **D)** Representative images of H&E staining of kidney (left panel), with representative images of human vimentin (green) to identify tumor and mouse CD31 (red) and to identify host vascular cells (right panel). Comparisons of CD31 staining of excised kidney in tumor (E) and non-tumor (F) regions following neoadjuvant sunitinib treatment. *Symbols and bars for box and whiskers plot:* median (line), upper/lower quartile (box), min/max (error bars). *Survival analysis:* based on Kaplan-Meier analysis. N=7-11 mice per group. * p<0.05 | ** p<0.01 compared to control.

Figure S8: Randomization and equal sorting of mice prior to neoadjuvant treatment. Prior to neoadjuvant therapy, tumor-bearing animals were sorted into groups of equal tumor burden

based on tumor volume derived from caliper measurements (breast and melanoma models) or on whole body BLI (for kidney models). Examples shown for **A)** Human Kidney model SN12-PM6^{LUC+}(See Figure 1A), **B)** Mouse Kidney model RENCA^{LUC+} (See Figure 1E), **C)** Human Melanoma model WM113/6-4L (See Figure S2A), and **D)** human breast model LM2-4^{LUC+} (see Figure 2A). BLI, Bioluminescence imaging

Table S1: Summary of Drugs and Dosing Schedules.

Drug Name	Drug Type and Target	Dose/Duration
**G6.31**	**Mouse/human VEGF neutralizing antibody**	5 mg/kg /3 days
**B.20**	**Mouse/human VEGF neutralizing antibody**	5 mg/kg/ 3 days
**DC101**	**Mouse VEGFR-2 neutralizing antibody**	800ug/ 3days
**CT322**	**Mouse/human VEGFR-2 neutralizing fibronectin mimic (adnectin)**	100 mg/kg /3 days
**OXI-5403**	**Vascular disrupting agent**	10 mg/kg/ 1x week or 50 mg/kg every 2 weeks
**Sunitinib**	**VEGF RTKI (c-kit, PDGFR, etc)**	60 mg/kg/day (14 Day schedule)<br>120 mg/kg/day (7 Day schedule)
**Axitinib**	**VEGF RTKI (c-kit, PDGFR, etc)**	100 mg/kg/day (14 Day schedule)
**LDM CTX/UFT**	**Low dose metronomic (LDM) cyclophosphamide (CTX) with 5-FU and Tegafur (UFT)**	CTX 20 mg/kg + UFT 15 mg/kg
**LDM CTX/VBL**	**Low dose metronomic (LDM) cyclophosphamide (CTX) with vinblastine (VBL)**	CTX 20 mg/kg + VBL 0.33 mg/kg
**MTD CTX**	**Maximum tolerated dose (MTD) cyclophosphamide (CTX)**	100 mg/kg 3/week
**Crizotinib**	**C-met/ALK TKI**	50 mg/kg/ day

Figure S1

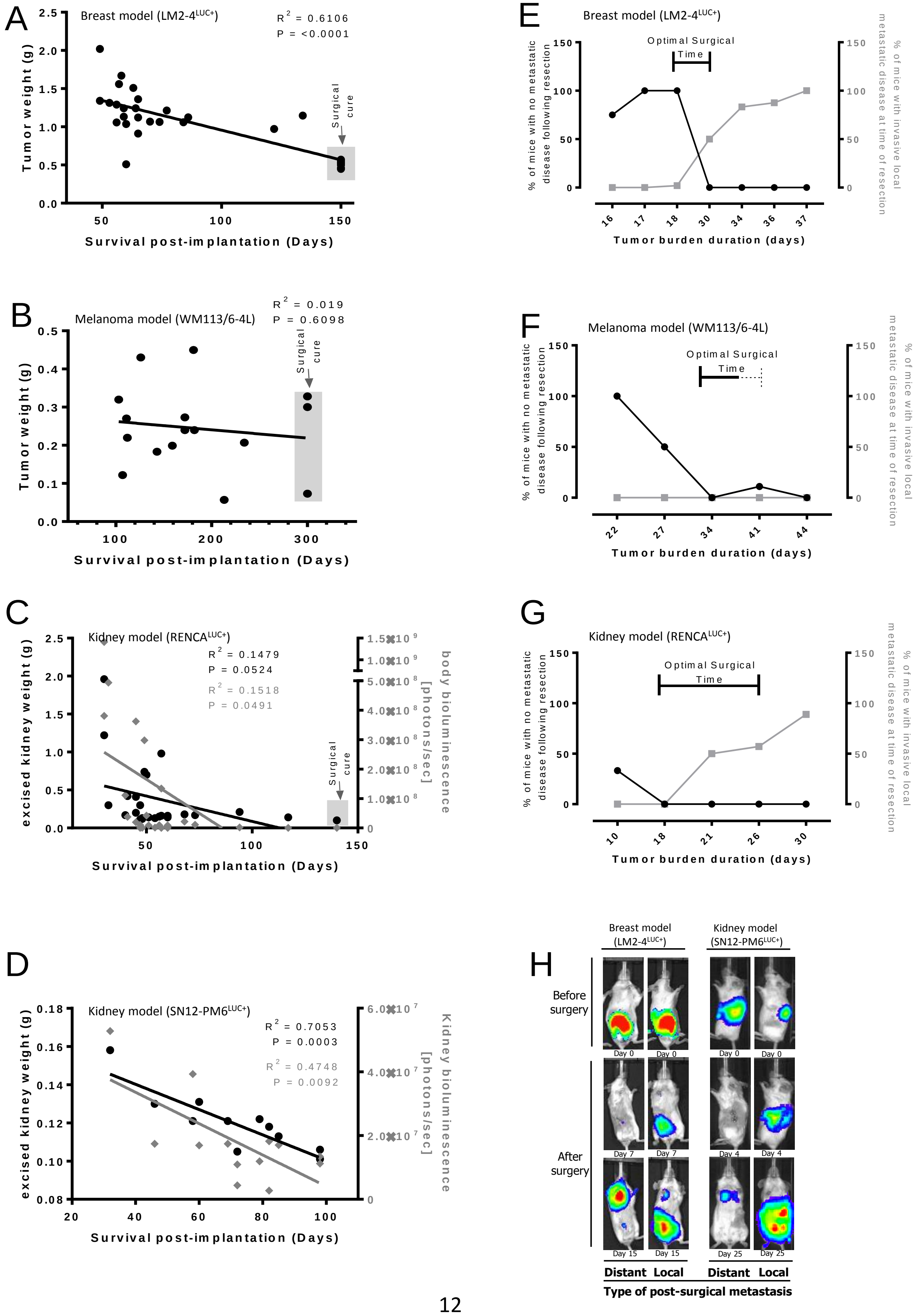
A
Breast model (LM2-4LUC+)
R2 = 0.6106
P = <0.0001
Surgical cure
Tumor weight (g)
Survival post-implantation (Days)
B
Melanoma model (WM113/6-4L)
R2 = 0.019
P = 0.6098
Surgical cure
Tumor weight (g)
Survival post-implantation (Days)
C
Kidney model (RENCALUC+)
R2 = 0.1479
P = 0.0524
R2 = 0.1518
P = 0.0491
Surgical cure
excised kidney weight (g)
body bioluminescence [photons/sec]
Survival post-implantation (Days)
D
Kidney model (SN12-PM6LUC+)
R2 = 0.7053
P = 0.0003
R2 = 0.4748
P = 0.0092
excised kidney weight (g)
Kidney bioluminescence [photons/sec]
Survival post-implantation (Days)
E
Breast model (LM2-4LUC+)
Optimal Surgical Time
% of mice with no metastatic disease following resection
% of mice with invasive local metastatic disease at time of resection
Tumor burden duration (days)
F
Melanoma model (WM113/6-4L)
Optimal Surgical Time
% of mice with no metastatic disease following resection
% of mice with invasive local metastatic disease at time of resection
Tumor burden duration (days)
G
Kidney model (RENCALUC+)
Optimal Surgical Time
% of mice with no metastatic disease following resection
% of mice with invasive local metastatic disease at time of resection
Tumor burden duration (days)
H
Breast model (LM2-4LUC+)
Kidney model (SN12-PM6LUC+)
Before surgery
After surgery
Day 0
Day 7
Day 4
Day 15
Day 25
Distant Local Distant Local
Type of post-surgical metastasis

Figure S2

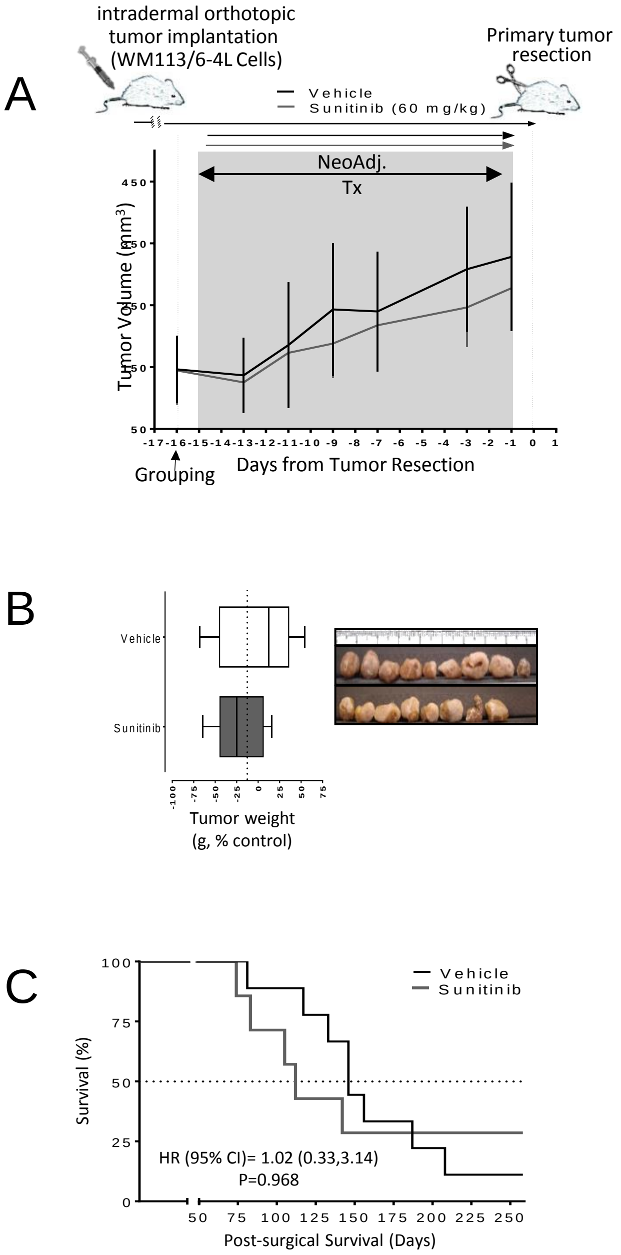
A
intradermal orthotopic tumor implantation (WM113/6-4L Cells)
Primary tumor resection
Vehicle
Sunitinib (60 mg/kg)
NeoAdj. Tx
Tumor Volume (mm³)
450
50
-17 -16 -15 -14 -13 -12 -11 -10 -9 -8 -7 -6 -5 -4 -3 -2 -1 0 1
Grouping
Days from Tumor Resection
B
Vehicle
Sunitinib
-100 -75 -50 -25 0 25 50 75
Tumor weight (g, % control)
C
100
75
50
25
0
Survival (%)
Vehicle
Sunitinib
HR (95% CI)= 1.02 (0.33,3.14)
P=0.968
50 75 100 125 150 175 200 225 250
Post-surgical Survival (Days)

Figure S3

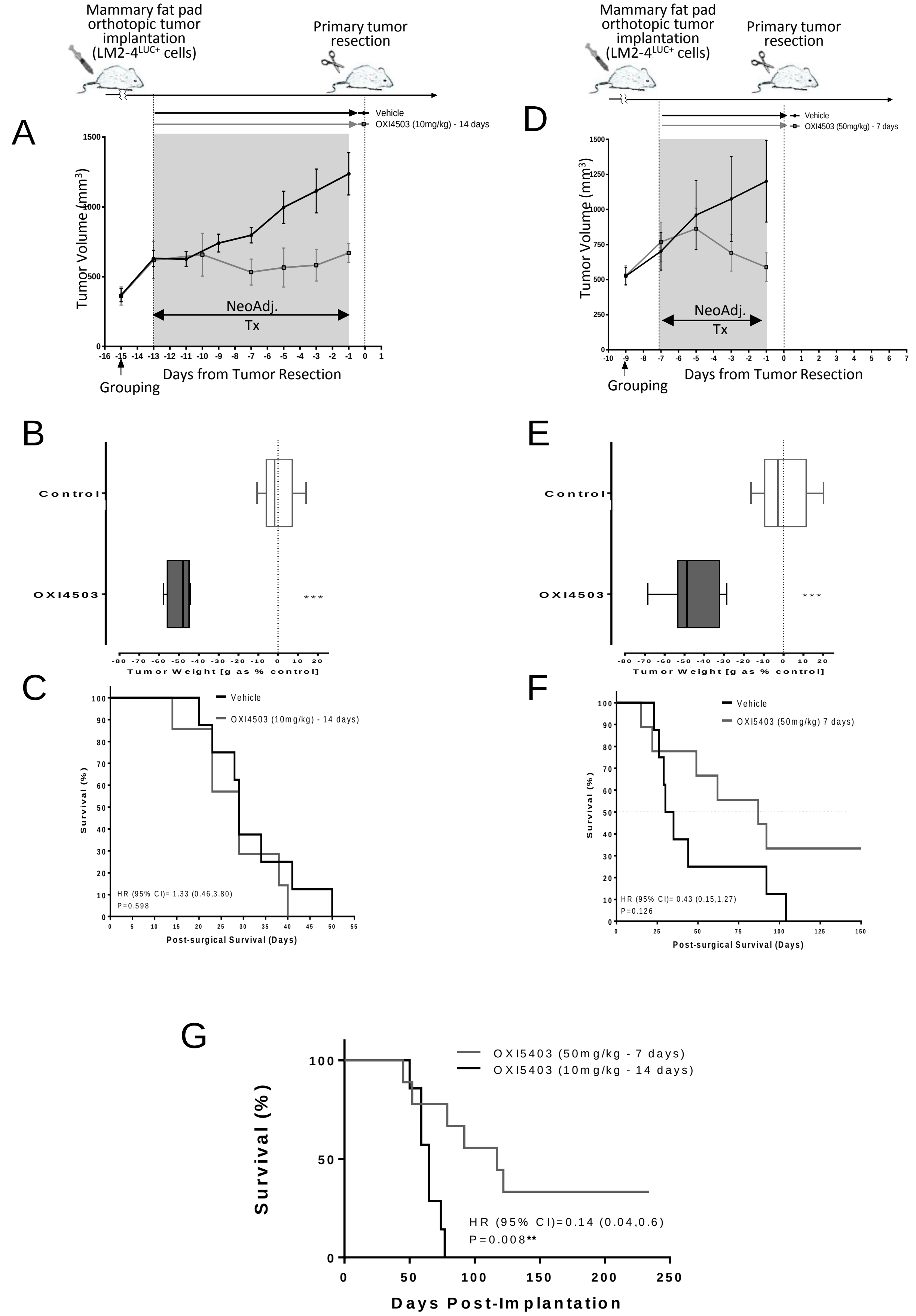

A
Mammary fat pad orthotopic tumor implantation (LM2-4LUC+ cells)
Primary tumor resection
Vehicle
OXI4503 (10mg/kg) - 14 days
Tumor Volume (mm3)
NeoAdj. Tx
Days from Tumor Resection
Grouping
B
Control
OXI4503
***
Tumor Weight [g as % control]
C
Vehicle
OXI4503 (10mg/kg) - 14 days
Survival (%)
HR (95% CI)= 1.33 (0.46,3.80)
P=0.598
Post-surgical Survival (Days)
D
Mammary fat pad orthotopic tumor implantation (LM2-4LUC+ cells)
Primary tumor resection
Vehicle
OXI4503 (50mg/kg) - 7 days
Tumor Volume (mm3)
NeoAdj. Tx
Days from Tumor Resection
Grouping
E
Control
OXI4503
***
Tumor Weight [g as % control]
F
Vehicle
OXI5403 (50mg/kg) 7 days
Survival (%)
HR (95% CI)= 0.43 (0.15,1.27)
P=0.126
Post-surgical Survival (Days)
G
OXI5403 (50mg/kg - 7 days)
OXI5403 (10mg/kg - 14 days)
Survival (%)
HR (95% CI)=0.14 (0.04,0.6)
P=0.008**
Days Post-Implantation

Figure S4

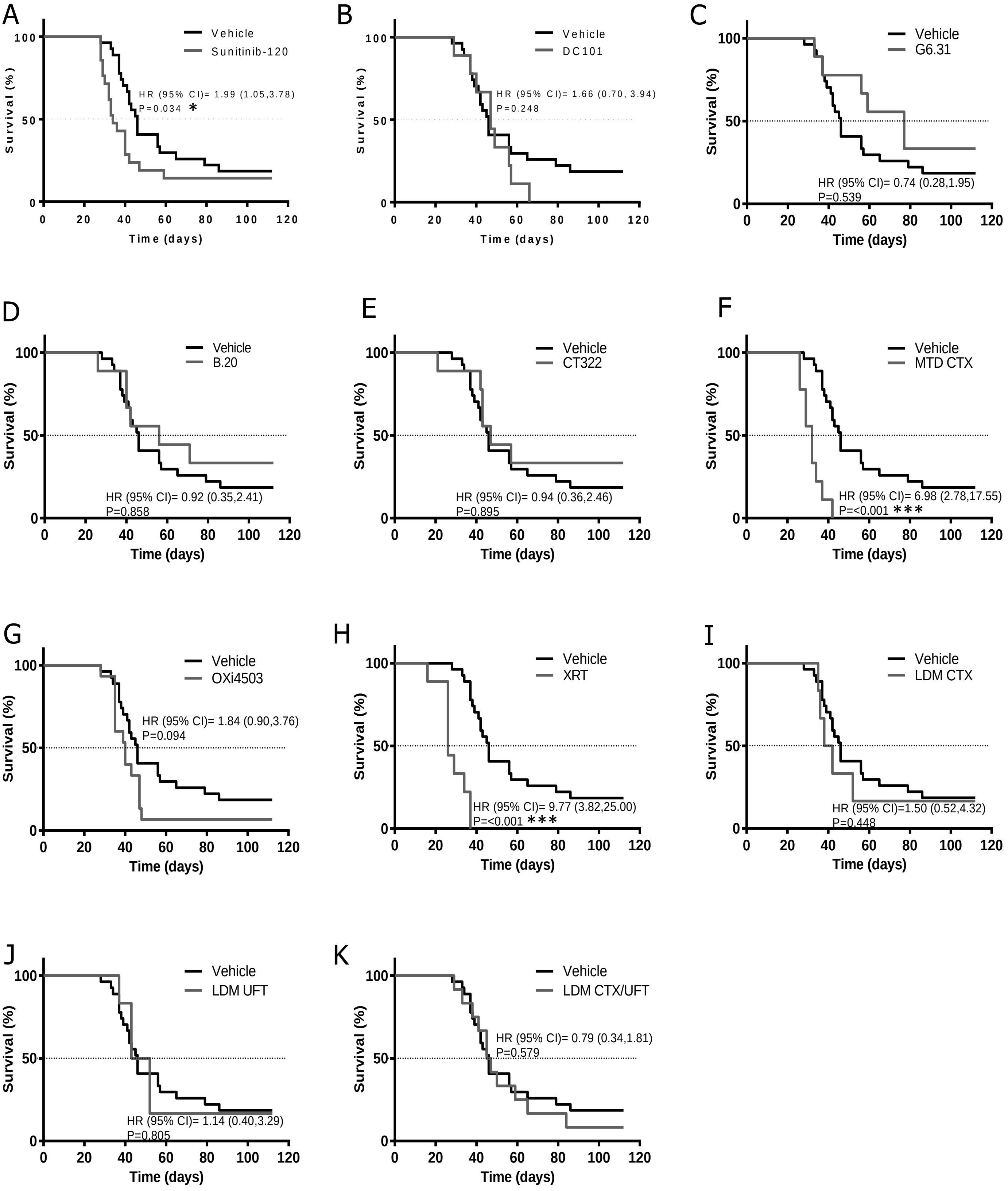

A
Vehicle
Sunitinib-120
HR (95% CI)= 1.99 (1.05,3.78)
P=0.034 *
Survival (%)
Time (days)
B
Vehicle
DC101
HR (95% CI)= 1.66 (0.70, 3.94)
P=0.248
Survival (%)
Time (days)
C
Vehicle
G6.31
HR (95% CI)= 0.74 (0.28,1.95)
P=0.539
Survival (%)
Time (days)
D
Vehicle
B.20
HR (95% CI)= 0.92 (0.35,2.41)
P=0.858
Survival (%)
Time (days)
E
Vehicle
CT322
HR (95% CI)= 0.94 (0.36,2.46)
P=0.895
Survival (%)
Time (days)
F
Vehicle
MTD CTX
HR (95% CI)= 6.98 (2.78,17.55)
P=<0.001 ***
Survival (%)
Time (days)
G
Vehicle
OXi4503
HR (95% CI)= 1.84 (0.90,3.76)
P=0.094
Survival (%)
Time (days)
H
Vehicle
XRT
HR (95% CI)= 9.77 (3.82,25.00)
P=<0.001 ***
Survival (%)
Time (days)
I
Vehicle
LDM CTX
HR (95% CI)=1.50 (0.52,4.32)
P=0.448
Survival (%)
Time (days)
J
Vehicle
LDM UFT
HR (95% CI)= 1.14 (0.40,3.29)
P=0.805
Survival (%)
Time (days)
K
Vehicle
LDM CTX/UFT
HR (95% CI)= 0.79 (0.34,1.81)
P=0.579
Survival (%)
Time (days)

Figure S5

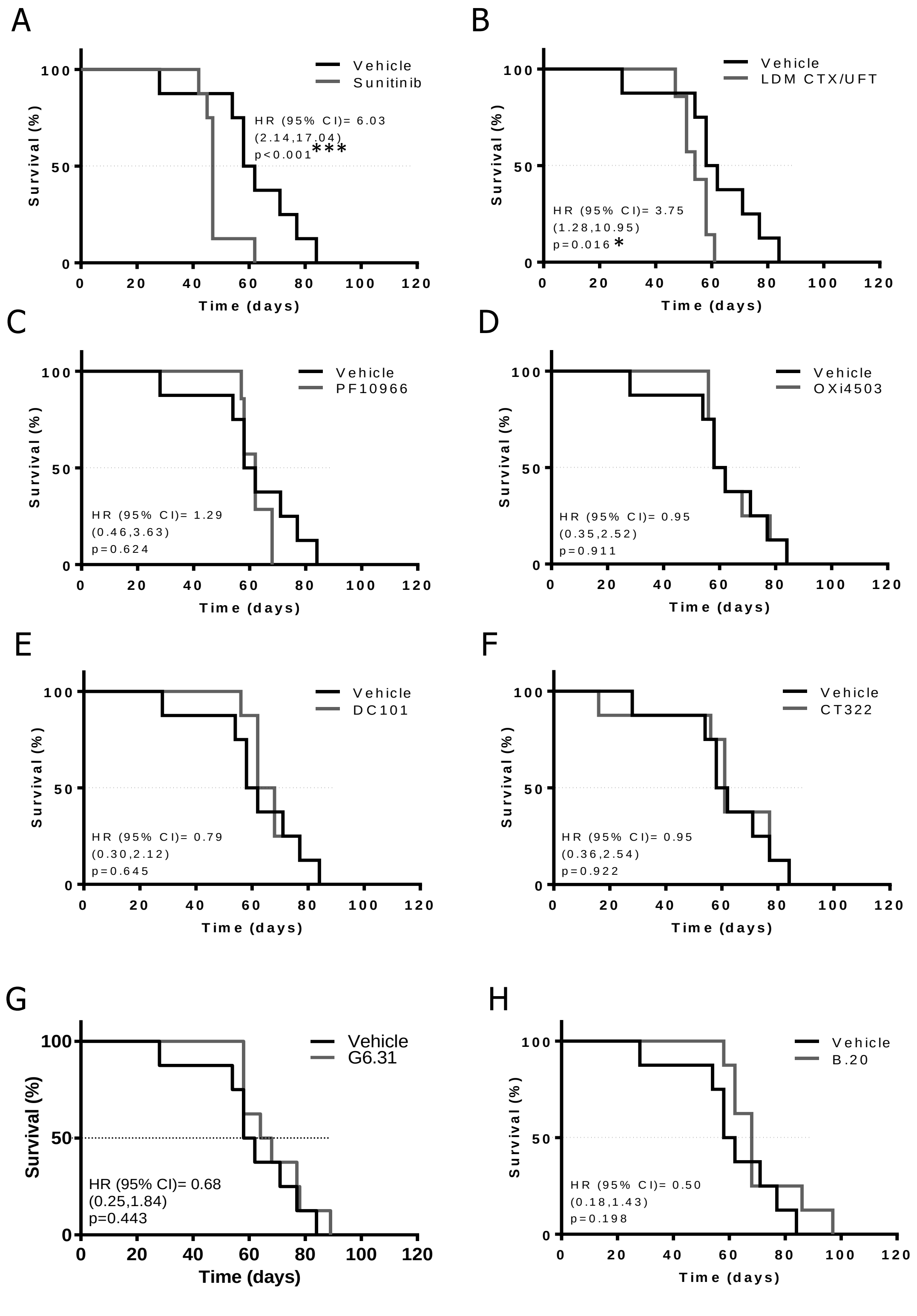
A
Vehicle
Sunitinib
HR (95% CI)= 6.03
(2.14,17.04)
p<0.001***
Survival (%)
Time (days)
B
Vehicle
LDM CTX/UFT
HR (95% CI)= 3.75
(1.28,10.95)
p=0.016*
Survival (%)
Time (days)
C
Vehicle
PF10966
HR (95% CI)= 1.29
(0.46,3.63)
p=0.624
Survival (%)
Time (days)
D
Vehicle
OXi4503
HR (95% CI)= 0.95
(0.35,2.52)
p=0.911
Survival (%)
Time (days)
E
Vehicle
DC101
HR (95% CI)= 0.79
(0.30,2.12)
p=0.645
Survival (%)
Time (days)
F
Vehicle
CT322
HR (95% CI)= 0.95
(0.36,2.54)
p=0.922
Survival (%)
Time (days)
G
Vehicle
G6.31
HR (95% CI)= 0.68
(0.25,1.84)
p=0.443
Survival (%)
Time (days)
H
Vehicle
B.20
HR (95% CI)= 0.50
(0.18,1.43)
p=0.198
Survival (%)
Time (days)

Figure S6

A

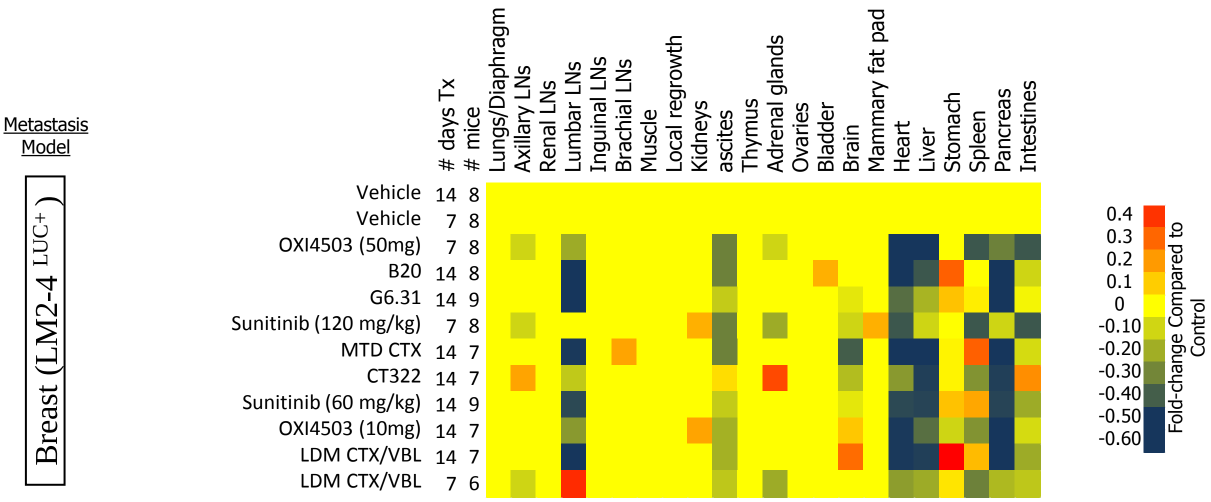
Metastasis Model
Breast (LM2-4 LUC+)
days Tx
mice
Lungs/Diaphragm
Axillary LNs
Renal LNs
Lumbar LNs
Inguinal LNs
Brachial LNs
Muscle
Local regrowth
Kidneys
ascites
Thymus
Adrenal glands
Ovaries
Bladder
Brain
Mammary fat pad
Heart
Liver
Stomach
Spleen
Pancreas
Intestines
Vehicle 14 8
Vehicle 7 8
OXI4503 (50mg) 7 8
B20 14 8
G6.31 14 9
Sunitinib (120 mg/kg) 7 8
MTD CTX 14 7
CT322 14 7
Sunitinib (60 mg/kg) 14 9
OXI4503 (10mg) 14 7
LDM CTX/VBL 14 7
LDM CTX/VBL 7 6
0.4
0.3
0.2
0.1
0
-0.10
-0.20
-0.30
-0.40
-0.50
-0.60
Fold-change Compared to Control

Figure S7

A

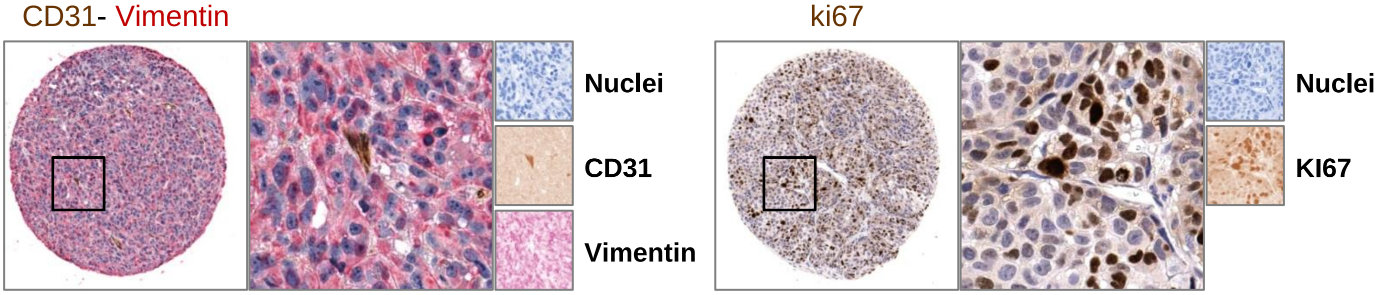

B

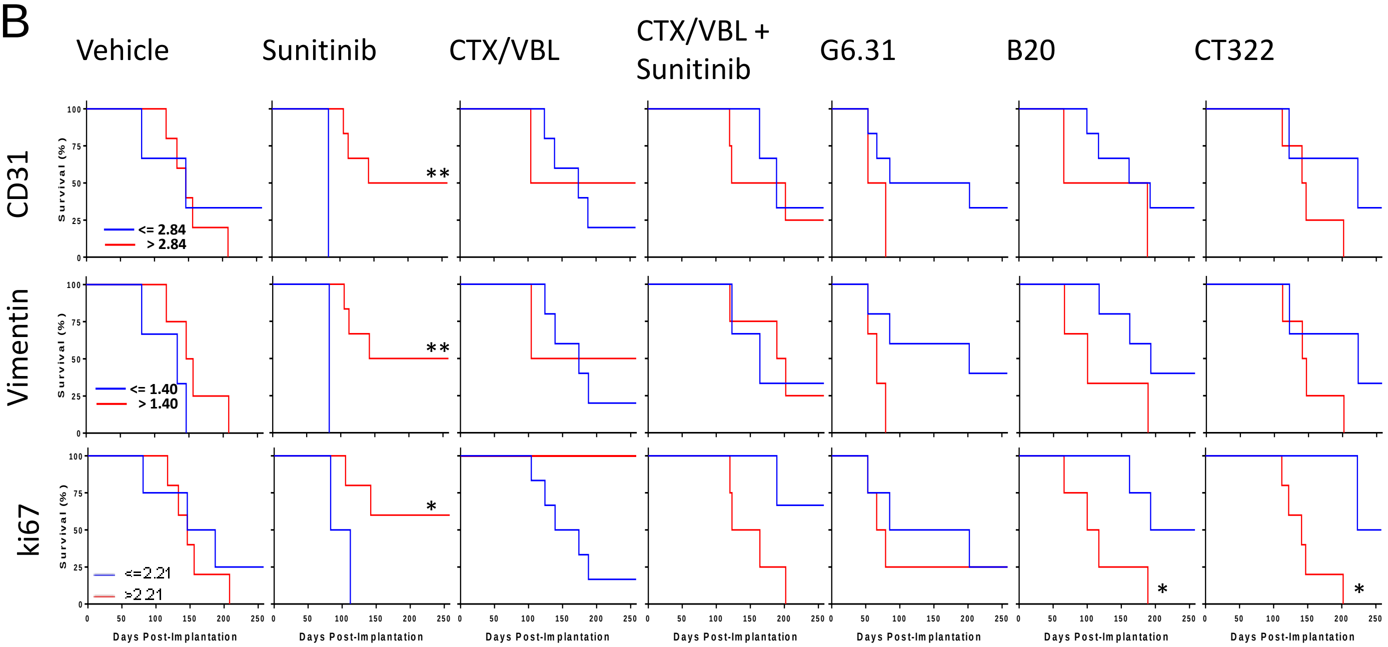

C

P-values	Vehicle	Sunitinib	CTX/VBL	CTX/VBL + Sunitinib	G6.31	B20	CT322
**CD31**	0.608	**0.008**	0.805	0.742	0.150	0.267	0.174
**Vimentin**	0.961	**0.008**	0.805	0.934	0.053	0.073	0.174
**Ki67**	0.501	**0.048**	0.321	0.069	0.654	**0.027**	**0.041**

D

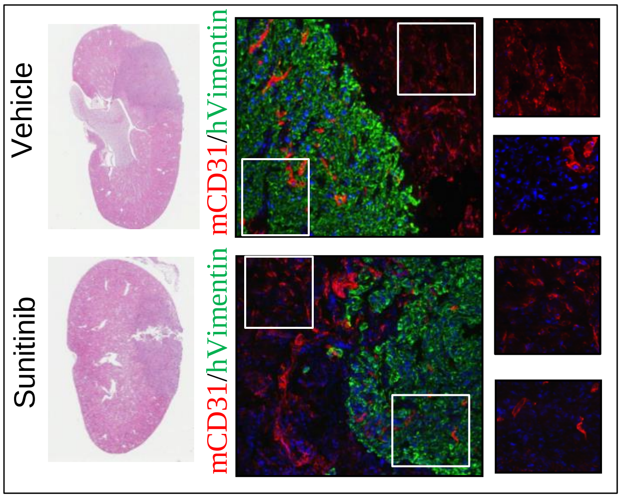

E

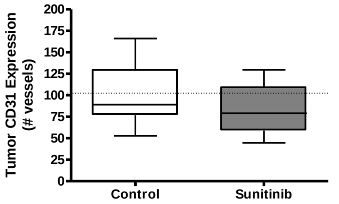

F

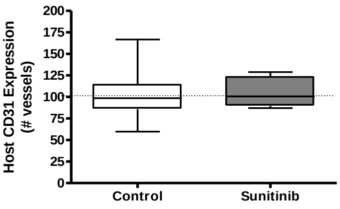

Figure S8

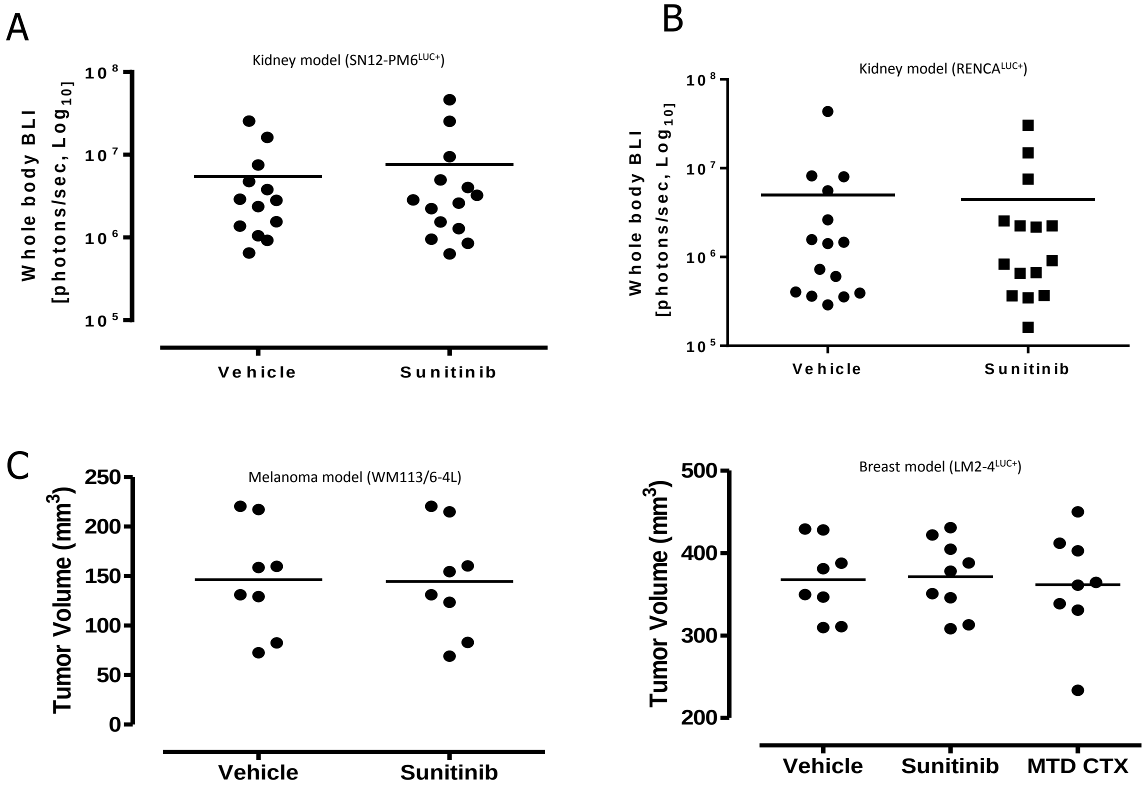
A
Kidney model (SN12-PM6LUC+)
Whole body BLI [photons/sec, Log10]
10 8
10 7
10 6
10 5
Vehicle
Sunitinib
B
Kidney model (RENCALUC+)
Whole body BLI [photons/sec, Log10]
10 8
10 7
10 6
10 5
Vehicle
Sunitinib
C
Melanoma model (WM113/6-4L)
Tumor Volume (mm3)
250
200
150
100
50
0
Vehicle
Sunitinib
Breast model (LM2-4LUC+)
Tumor Volume (mm3)
500
400
300
200
Vehicle
Sunitinib
MTD CTX

Reference List

Cruz-Munoz W, Man S, Xu P, Kerbel RS (2008) Development of a preclinical model of spontaneous human melanoma central nervous system metastasis. Cancer Res 68**:** 4500-4505

Ebos JM, Lee CR, Bogdanovic E, Alami J, Van Slyke P, Francia G, Xu P, Mutsaers AJ, Dumont DJ, Kerbel RS (2008) Vascular endothelial growth factor-mediated decrease in plasma soluble vascular endothelial growth factor receptor-2 levels as a surrogate biomarker for tumor growth. Cancer Res 68**:** 521-529

Ebos JM, Lee CR, Christensen JG, Mutsaers AJ, Kerbel RS (2007) Multiple circulating proangiogenic factors induced by sunitinib malate are tumor-independent and correlate with antitumor efficacy. Proc Natl Acad Sci U S A 104**:** 17069-17074

Ebos JM, Lee CR, Cruz-Munoz W, Bjarnason GA, Christensen JG, Kerbel RS (2009) Accelerated metastasis after short-term treatment with a potent inhibitor of tumor angiogenesis. Cancer Cell 15**:** 232-239

Fidler IJ, Naito S, Pathak S (1990) Orthotopic implantation in essential for the selection, growth and metastasis of human renal cell cancer in nude mice. Cancer Metastasis Rev 9**:** 145-165

Kato Y, Yoshimura K, Shin T, Verheul H, Hammers H, Sanni TB, Salumbides BC, Van Erp K, Schulick R, Pili R (2007) Synergistic in vivo antitumor effect of the histone deacetylase inhibitor MS-275 in combination with interleukin 2 in a murine model of renal cell carcinoma. Clin Cancer Res 13**:** 4538-4546

Man S, Bocci G, Francia G, Green S, Jothy S, Bergers G, Hanahan D, Bohlen P, Hicklin DJ, Kerbel RS (2002) Antitumor and anti-angiogenic effects in mice of low-dose (metronomic) cyclophosphamide administered continuously through the drinking water. Cancer Res 62**:** 2731-2735

Munoz R, Man S, Shaked Y, Lee C, Wong J, Francia G, Kerbel RS (2006) Highly efficacious non-toxic treatment for advanced metastatic breast cancer using combination UFT-cyclophosphamide metronomic chemotherapy. Cancer Res 66**:** 3386-3391

Tracz A, Mastri M, Lee CR, Pili R, Ebos JM (2014) Modeling spontaneous metastatic renal cell carcinoma (mRCC) in mice following nephrectomy. Journal of visualized experiments : JoVE
